# A robust absolute quantification protocol for potato virus Y based on reverse transcription-quantitative PCR

**DOI:** 10.17912/micropub.biology.001779

**Published:** 2025-10-22

**Authors:** Diana Spencer, Elizabeth Padilla-Crespo, Ek Han Tan

**Affiliations:** 1 School of Biology and Ecology, University of Maine, Orono, Maine, United States; 2 InterAmerican University of Puerto Rico, Aguadilla, Puerto Rico

## Abstract

Standardized protocols for absolute quantification of potato virus Y (PVY) from potato tissue is critical for host-virus dynamic studies. Here, we developed a standardized protocol using a cloned viral sequence as standards to detect and quantify PVY. Starting with total RNA, concentrated via column-based kit, this protocol is able to detect approximately 50 viral copies/reaction from multiple PVY strains. Validation of this protocol confirmed linearity across 8 orders of magnitude with high repeatability, reproducibility and statistical robustness across three independent runs. This protocol offers reliable PVY quantification to manage potato crop health and enables comparative studies with other viral systems.

**Figure 1. Absolute quantification of potato virus Y (PVY) by reverse transcription-quantitative PCR provides a robust, reproducible framework for assessing PVY viral load:**
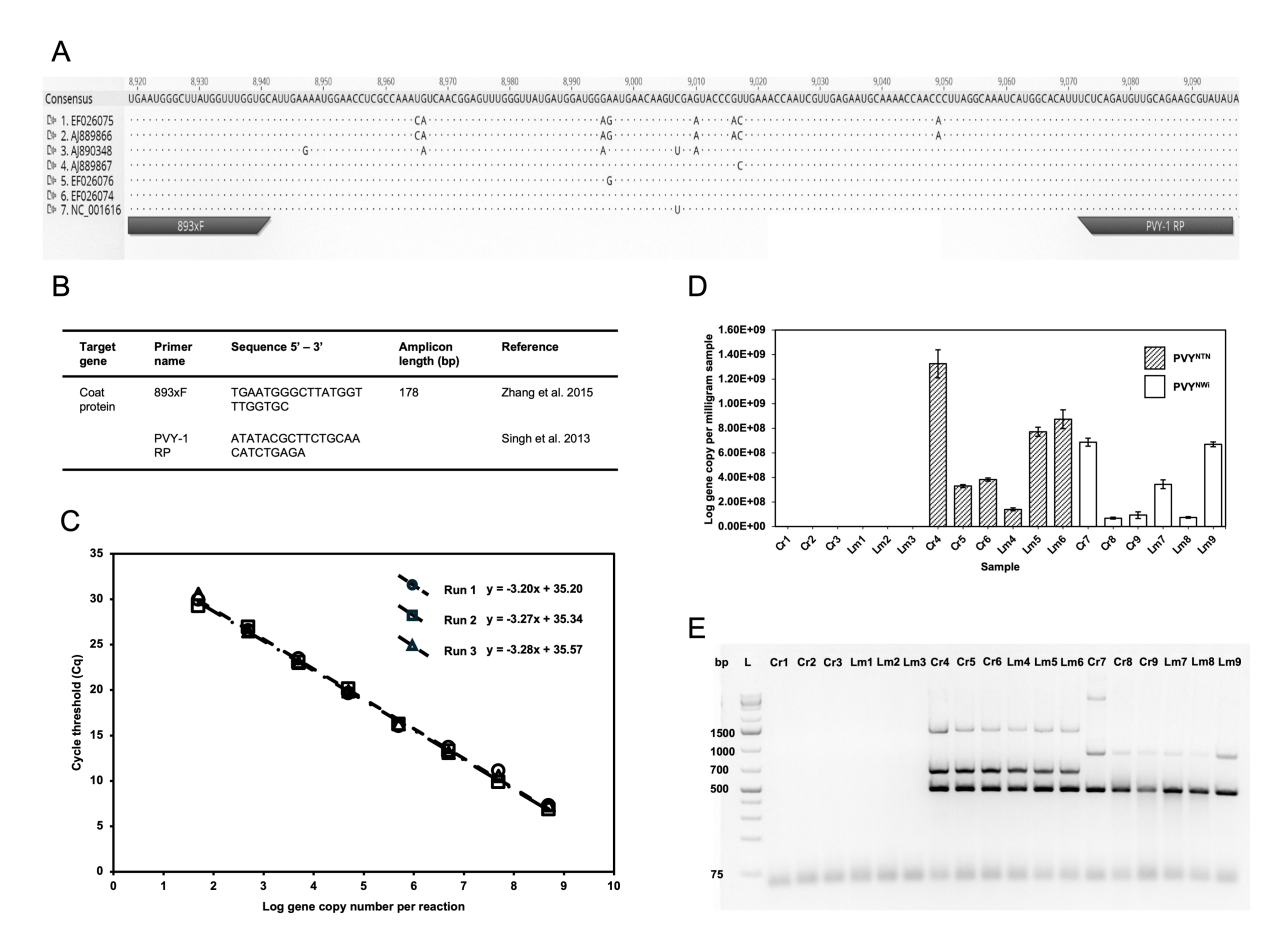
(A) Alignment of primers 893xF and PVY-1 RP on six PVY strains. The strains listed are PVY ^NTNa^ (EF026075), PVY ^NTNb^ (AJ889866), PVY ^C^ (AJ890348), PVY ^NWib^ (AJ889867), PVY ^N:O^ (EF026076) and PVY ^O^ (EF026074). (B) Primers used for this PVY RT-qPCR protocol. (C) Standard curves plotted from three independent qPCR runs, each performed in triplicate using pPVYhvedi1, a cloned PVY viral plasmid for absolute quantification. Regression equations from each run are shown on the figure, and qPCR efficiency was observed at 101.6 – 105.4%, with R ^2^ values between 0.995 – 0.998. The plasmid concentration ranged 1 x 10 ^-9^ – 1 x 10 ^-16^ g/μL, corresponding to ~49 x 10 ^7^ to 49 gene copy numbers per reaction. (D) Bar plot showing absolute quantification of PVY viral copy number per miligram tissue using RT-qPCR from uninfected and infected Caribou Russet (Cr) and Lamoka (Lm). Cr1 to Cr3 and Lm1 to Lm3 are uninfected samples, Cr4 to Cr6 and Lm4 to Lm6 are PVY ^NTNa^ infected samples and Cr7 and Cr9 and Lm7 to Lm9 are PVY ^NWib^ infected samples. Error bars show the standard deviation from the three technical replicates from each sample. (E) Gel electrophoresis image of multiplex RT-PCR confirmation of uninfected (no bands) and infected samples with DNA ladder (L) using the protocol from Chikh Ali et al. (2010) as described in the methods section. PVY ^NTNa^ had the expected amplicons of 1307, 633 and 441 bp and PVY ^NWib^ had the expected amplicons of 853 and 441 bp.

## Description

Potato virus Y (PVY) is a single-stranded positive strand RNA virus that negatively impacts potato production, and remains the most prevalent potato virus globally (Karasev and Gray, 2013; Kreuze et al., 2019; Lacomme et al., 2017). Multiple strains of PVY impact the potato industry but increasingly, recombinant PVY strains have become prominent in potato growing regions (Green et al., 2017; Karasev et al., 2008; Karasev et al., 2011). PVY infected potato results in an unfavorable outlook for plant health, vigor, yield and reduces tuber quality. Losses due to PVY are dependent on multiple factors such as virus strain and potato variety, but between 10 – 100% yield reduction have been observed (Tsedaley, 2015). In the field, PVY spreads easily in a non-persistent manner by aphids and can also be transmitted mechanically through leaf injuries.

Common practices to detect PVY by potato growers are by visual symptoms, by commercial ELISA kits, or by rapid test kits (Lacomme and Jacquot, 2017). Due to the nature of PVY as an RNA virus, reverse transcription (RT)-PCR has also been used for its detection. This nucleic acid-based assay requires RNA isolation and reverse transcription to synthesize complementary DNA (cDNA), followed by either by bands on a gel-based conventional PCR or by quantitative PCR (qPCR) (Chikh Ali et al., 2010; MacKenzie el at., 2015; Singh et al., 2013). The use of bands on a gel-based conventional PCR is useful for differentiating PVY strains after RNA conversion to cDNA, and the multiplex PCR assay developed by Chikh Ali et al. (2010) is used widely for infection status or strain identification. For reverse transcription-quantitative PCR (RT-qPCR), two methods can be employed: one-step RT-qPCR, in which the RT and qPCR reactions are perfomed in a single tube; or two-step RT-qPCR, where cDNA synthesis is perfomed separetely, and an aliquot of cDNA is subsequently used as template for the qPCR (Khelifa, 2019; Davie et al., 2017). Khelifa (2019) developed a one-step RT-qPCR protocol with absolute quantification using standards derived from RNA-based in vitro transcription. This method is technically challenging, requiring direct in vitro transcription and proper RNA storage prior to performing any experiments. A two-step RT-qPCR method was developed by Davie et al. (2017) based on the relative quantification 2 ^-ddCt^ method, but is not suited for absolute virus titering because it's based on relative changes in gene expression. Both Khelifa (2019) and Davie et al. (2017) used the TaqMan probe-based qPCR system. SYBR Green-based qPCR can also be utilized to study PVY, such as the one-step RT-qPCR assay that was developed by Zhang et al. (2015) for PVY detection. Based on our literature search, a SYBR Green-based RT-qPCR protocol for absolute quantitation of PVY has not yet been published, which we report here.

We developed this two-step RT-qPCR using absolute quantification using a cloned viral plasmid (DNA-based) with SYBR Green chemistry for PVY in order to expand the molecular toolkit for PVY studies in potato. In other plant viral systems, DNA-based absolute quantification method has been successfully implemented using cloned viral sequences (Bashir et al., 2022; Kollenberg et al., 2014; Shirima et al., 2017; Simón et al., 2018). This protocol was also developed as part of an integration workflow for the Host-Virus Dynamics Institute (HVEDI), where many disparate viral systems are being studied. Within HVEDI, absolute quantification qPCR or RT-qPCR and the use of SYBR Green chemistry were standardized as the integrated molecular method to determine viral loads across the disparate viral systems from all domains of life (Bahramian et al., 2022; Ceballos et al., 2020; Hour et al., 2025). SYBR Green chemistry was selected due to cost-effectiveness for single gene targets and the fact that it does not require TaqMan probe design. In addition, a cloned viral plasmid (which will be different for each virus system) was used as the substrate for the derivaton of the standard curves to perform absolute quantification for each HVEDI viral system. During protocol development, we also considered the applicability of this to facilitate tissue and virus collection, ensuring consistent results from greenhouse to field conditions (Hamim et al., 2022; Wille et al., 2018; Yockteng et al., 2013). This led to the development of this protocol that starts with the collection of 6 mm leaf discs in RNA *later* ™ for preservation, and an RNA purification and concentration method that allows for the detection of PVY-infected potato from total RNA.

Two established protocols for PVY RT-qPCR were consulted and we evaluated two primer pairs: 893xF/903xR (Zhang et al., 2015) which produces a 130 bp amplicon, and the primer pair PVY-1 FP/PVY-1 RP (Singh et al., 2013) which produces a 74 bp amplicon. Since larger PCR amplicons are preferable to smaller amplicons for viral RNA integrity and viability estimations (Van Holm et al., 2021), the combination consisting of primers 893xF and PVY-1 RP which produces a larger amplicon size of 178 bp was selected. Primer specificity against different PVY strains was checked via Primer-BLAST (Ye et al., 2012) under broad-coverage search settings, and by visual examination with a multiple sequence alignment using Geneious (version 11.1.5). Both 893xF and PVY-1 RP exhibited high specificity, aligning to their respective target region (positions 8919–8941 and 9071–9096) on the Coat Protein (CP) gene across six PVY strains, PVY ^NTNa^ (EF026075), PVY ^NTNb^ (AJ889866), PVY ^C^ (AJ890348), PVY ^NWib^ (AJ889867), PVY ^N:O^ (EF026076) and PVY ^O^ (EF026074), with no observed mismatches ( Figure 1A and Figure 1B ). Primer-BLAST analysis indicated that this primer pair matched all PVY strains infecting potato with 100% sequence identity. Although primer 893xF aligned to the plum pox virus genome (Maiss et al., 1989), the reverse primer PVY-1 RP did not, and thus will not amplify this genome. These findings confirm that the primer set is highly specific and effective for targeting the intended region across multiple PVY strains.

We cloned the 178 bp PVY CP gene amplicon into a pCR™Blunt II-TOPO™ plasmid vector, to create the pPVYhvedi1 plasmid. The qPCR performance and efficiency using pPVYhvedi1 was tested by generating qPCR standard curves over three independent runs ( Figure 1C ). The ideal qPCR slope is within -3.1 and -3.6, corresponding to an efficiency range of 90% to 110%, where a 100% efficiency signifies that the PCR product quantity doubles with each amplification cycle. In our assays using pPVYhvedi1, the standard curves showed slopes ranging from -3.20 to -3.28 and amplification efficiencies from 101.6% to 105.5%, indicating optimal amplification kinetics (Rogers-Broadway and Karteris, 2015). The assay covered a broad dynamic range, with the pPVYhvedi1 plasmid concentrations spanning from 1 × 10 ^-9^ to 1 × 10 ^-16^ g/μL, ensuring reliable quantification across multiple orders of magnitude. The limit of detection (LOD) for the pPVYhvedi1 standard curve was determined to be at 10 ^-16^ g/μL, corresponding to 49 gene copies within a 2 μL template volume in the qPCR assay. One-way analysis of variance (ANOVA) of the standard curves yielded a *p* -value > 0.05, indicating no significant difference among the standard curves across the three runs.

Following validation of the qPCR assay using pPVYhvedi1, quantification of PVY from infected and uninfected potato leaves were performed using purified RNA from leaf discs stored in RNA *later* ™, concentrated with the Zymo Direct-zol™ RNA Microprep Kit. Before RNA extraction, each leaf disc was weighed on a precision scale after being blotted dry. Uninfected, PVY ^NTNa^ -infected and PVY ^NWib^ -infected Caribou Russet and Lamoka were used in this experiment. Uninfected Caribou Russet and Lamoka had “undetermined” C _q_ values, resulting in zero viral copies as well as no amplification from multiplex RT-PCR ( Figure 1D and 1E). From this experiment, the gene copy number of PVY ^NTNa^ per milligram of sample ranged from 1.40 x 10 ^8^ to 1.33 x 10 ^9^ , whereas PVY ^NWib^ gene copy numbered from 6.88 x 10 ^7^ to 6.88 x 10 ^8^ per milligram of sample across the two potato varieties. The PVY strains infecting each potato variety were also confirmed by bands on a gel-based conventional multiplex PCR using the same 1st strand cDNA mixture used for qPCR analyses ( Figure 1E ). As expected, no amplification was observed for uninfected samples Cr1-3 and Lm1-3, PVY ^NTNa^ was detected from samples Cr4-6 and Lm4-6, and PVY ^NWib^ was detected from samples Cr7-9 and Lm7-9.

Together, these results demonstrate the protocol’s effectiveness for detecting and quantifying PVY from potato infected with different PVY strains. This protocol also incorporates viral RNA stabilization within a single 6 mm leaf disc via storage in RNA *later* ™, making tissue collection from the field feasible. The long-term stability of viral RNA in RNA *later* ™ has ensured successful quantification of PVY from field samples even after one year of storage. The use of a column-based total RNA purification protocol also ensured adequate RNA concentration from the leaf disc for first-strand cDNA synthesis, PVY detection via multiplex RT-PCR and quantification of PVY via RT-qPCR from diluted cDNA. This method is more user-friendly and cost-effective compared to immunocapture IC-RT-PCR or RNA concentration by differential centrifugation (Chikh-Ali et al., 2013; Zhang et al., 2015).

RT-qPCR assays have been widely utilized for detecting PVY, but their application for absolute quantification to estimate PVY viral load have been less common (Agindotan et al., 2007; Rupar et al., 2013; Schumpp et al., 2021; Singh et al., 2013; Zhang et al., 2015). Most RT-qPCR assays developed for PVY quantification have relied on the TaqMan probe-based chemistry rather than SYBR Green-based chemistry (Alves et al., 2024; Davie et al., 2017; Khelifa, 2019). In this study, we developed a SYBR Green-based, two-step RT-qPCR assay for absolute quantification of PVY, providing an alternative to TaqMan-based methods. Unlike previous absolute quantification studies that used RNA-based standards for PVY (Khelifa, 2019), we employed DNA standards using the pPVYhvedi1 plasmid containing a cloned viral biomarker gene (in this case, the PVY Coat Protein gene). This approach simplified standard curve preparation and analysis by eliminating the need for preparing the RNA-based standards. The use of a plasmid standard also enhances the reproducibility and consistency of viral quantification across different runs by ensuring stable and consistent calibration curves across experiments. Our PVY RT-qPCR primer set targets multiple PVY strains, therefore providing a universal method to quantify current PVY strains. Finally, this PVY quantification protocol was shown to be robust, providing a reproducible framework for assessing viral load and enabling improved evaluation of host-virus dynamics.

## Methods

**Uninfected and PVY-infected potato samples**

Uninfected and PVY-infected potato tubers ( *Solanum tuberosum* L.) from varieties Caribou Russet and Lamoka were obtained from Aroostook Farm in Presque Isle, Maine from the 2022 growing season. Uninfected and infected tubers were selected from mother plants that had been genotyped by multiplex RT-PCR (Chikh Ali et al., 2010) to confirm the absence or presence of single strain infections with PVY ^NTNa^ or PVY ^NWib^ in each each variety. Uninfected and infected tubers were planted individually in a one-gallon pot in a cage with mesh walls and a top, measuring 16 x 16 x 24 inches. These cages were grown at 24°C with 16-hour day length provided by supplemental lighting at the Roger Clapp Greenhouse at the University of Maine.

**Potato leaf disc collection and preservation**

Three leaflets were sampled from each potato plant at the fourth week post-plantation, with each sample measuring 6 mm in diameter, obtained using a single-hole punch. The single-hole punch was disinfected with 70% ethanol between sample collections to prevent contamination. Samples were individually immersed in 250 μL of RNA *later* ™ Stabilization Solution and incubated overnight at room temperature before storage at -20°C. Prior to RNA extraction, samples were removed from the solution, gently blotted dry and weighed.

**Total RNA extraction, concentration and reverse transcription.**

Samples were homogenized in 1.5 mL Safe-Lock tubes (Eppendorf, cat. no. 022363204), each containing 450 μL of TRIzol™ Reagent and a 3.2 mm stainless steel bead (Next Advance). Tissue homogenization was performed in a Bullet Blender (Next Advance) at high speed for 5 minutes, followed by centrifugation for 5 minutes at 4°C in a refrigerated centrifuge. 400 μL supernatants were transferred to new 1.5 mL nuclease-free tubes (USA Scientific, cat. no. 1615-5510). Total RNAs were subsequently extracted and purified using the Direct-zol™ RNA Microprep Kit following the manufacturer’s protocol, including DNase treatment steps, and eluted in 15 μL of DNase/RNase-free water.

The resulting RNA were subjected to first-strand cDNA synthesis using the SuperScript ^TM^ IV First-Strand Synthesis System according to the manufacturer’s protocol with several modifications described here. For primer annealing, 1 μL each of 50 μM Oligo d(T) primer, random hexamer and 10 mM dNTP mix, and water were combined with 1 μg of total RNA, resulting in a final reaction volume of 13 μL. The reactions were heated to 65°C for 5 minutes, followed by immediate cooling on ice for at least 1 minute. The reverse transcription (RT) reaction was prepared by adding 4 μL of 5x SSIV buffer and 1 μL each of 100 mM DTT, ribonuclease inhibitor, and reverse transcriptase (200 U/μL) to the annealed RNA mixture, yielding a total reaction volume of 20 μL. The reactions were incubated at 50°C for 45 minutes followed by heat inactivation at 80°C for 10 minutes to terminate the synthesis of first-strand cDNAs. The converted cDNAs were diluted to 1:20 in DNase/RNase-free water for qPCR analysis.

**Primers**

The forward and reverse primers used targeted the sequence of the conserved region of PVY coat protein gene ( Figure 1B ). This primer set was utilized to construct the PVY qPCR standards and amplify the region of interest in the PVY qPCR assay. Primer specificity was verified using Geneious (version 11.1.5) and Primer-BLAST (Ye et al., 2012) under broad-coverage search setting.

**Multiplex RT-PCR**

A PCR reaction containing 1 μL of first-strand cDNA (10 ng), 12.5 μL NEBNext ^®^ Q5 ^®^ Hot Start HiFi PCR Master Mix, a primer cocktail containing 12 oligos as decribed by Chikh Ali et al., (2010) and nuclease-free water to a final volume of 25 μL. PCR amplification was performed in a T100 thermal cycler (Bio-Rad) using the recommended cycling protocol by Chikh Ali et al., (2010). The PCR amplification products were separated on a 2.5% TAE gel, stained with ethidium bromide and visualized under UV illumination.

**Preparation of cloned PVY viral DNA standard**

The cloned PVY viral standard, pPVYhvedi1 (Addgene #23464), was generated by PCR amplification using forward primer 893xF and reverse primer PVY-1 RP producing 178 bp amplicons (Singh et al., 2013; Zhang et al., 2015). The PCR reaction contained 1 μL of first-strand cDNA (10 ng), 12.5 μL NEBNext ^®^ Q5 ^®^ Hot Start HiFi PCR Master Mix, primers and nuclease-free water to a final volume of 25 μL. Amplification was performed in a T100 thermal cycler (Bio-Rad) with the following thermal cycling parameters: initial denaturation at 95°C for 5 minutes, followed by 40 cycles of denaturation at 95°C for 15 seconds, and annealing and extension at 60°C for 1 minute. After amplification, 5 μL of the PCR product was resolved on a 1.5% agarose gel and the amplicons were purified using the freeze-and-squeeze method by incubating the extracted piece of agarose gel containing the amplicons in a -80°C freezer overnight. Subsequently, 2 μL of purified amplicons were cloned into the pCR™Blunt II-TOPO™ plasmid and transformed into competent *E. coli* following the manufacturer recommendations. Confirmation of pPVYhvedi1 insert sequence from the selected clone was performed using M13F and M13R primers by Sanger sequencing. Plasmids containing the targeted region were then purified using Zyppy ^TM^ Plasmid Miniprep Kit according to the manufacturer’s protocol.

**Absolute quantification qPCR assay for PVY**

Following reverse transcription, cDNA was subjected to SYBR Green-based qPCR using QuantStudio™ 3 Real-Time PCR System (Thermo Fisher Scientific) with three technical replicates. Each reaction mixture consisted of 2 μL template, 10 μL Power SYBR™ Green PCR Master Mix, 250 nM forward and reverse primers, and nuclease-free water, for a total volume of 20 μL. A series of absolute quantification standards using pPVYhvedi1 was prepared through a 1:10 serial dilution, starting from 1 x 10 ^-9^ g/μL down to 1 x 10 ^-16^ g/μL. The assay employed the standard curve with melt settings, with ROX as the passive reference and the thermocycling parameters are as follow: holding-stage at 95°C for 10 minutes; 40 cycles of PCR-stage set to 95°C for 15 seconds and 60°C for 60 seconds; followed by the standard QuantStudio™ 3 melt curve analysis. Quantification cycles (C _q_ ) were generated by the Design and Analysis 2 (DA2) software (Thermo Fisher Scientific, version 2.8.0).

**Calculation of PVY RNA copy number**

A standard curve was used to quantify the gene copy number of viral RNAs, which served as a proxy for the PVY count per milligram of sample. A standard curve was constructed by plotting the C _q_ values of the standard dilution series against their respective gene copy number. The gene copy number was calculated using Equation 1 (Ritalahti et al., 2006) where, 660 g/mol is the average molecular weight per base pair of double-stranded DNA and 6.023 x 10 ^23^ is Avogadro’s number.

Equation 1:

$$\mathrm{gene\&nbsp;copies}=\mathrm{DNA\&nbsp;concentration}\frac{\text{ng}}{\text{μL}}\times \frac{1\text{\&nbsp;g}}{1000^{3}\text{\&nbsp;ng}}\times \frac{1\text{\&nbsp;mol\&nbsp;bp\&nbsp;DNA}}{660\text{\&nbsp;g\&nbsp;DNA}}\times \frac{6.023\times 10^{23}\text{\&nbsp;bp}}{\text{mol\&nbsp;bp}}\times \frac{1\text{\&nbsp;copy}}{\text{plasmid\&nbsp;size\&nbsp;(bp)}}\times \text{volume\&nbsp;of\&nbsp;template}(\text{μL})$$

The number of target gene copies per milligram of sample was obtained using Equation 3. In this equation, the number of gene copy per reaction was extrapolated from the standard curve based on the reaction’s C _q_ value and the total volume of cDNA was calculated using Equation 2.

Equation 2:

$$\text{total volume of cDNA }(\mu L)=\frac{\text{volume of extracted RNA }(\mu L)}{\text{volume of RNA used }(\mu L)}\times \frac{\text{volume of first-strand cDNA synthesized }(\mu L)}{\text{volume of cDNA used for 1:20 dilution }(\mu L)}\times \frac{\text{volume of 1:20 cDNA }(\mu L)}{\text{volume of cDNA used per reaction }(\mu L)}$$

Equation 3:

$$\text{gene copies per mg sample = }\frac{\left(\text{gene copies per reaction × 2})\times \text{ total volume of cDNA }(\mu L\right)}{\text{weight of sample }(\mathrm{mg})}$$

The copy number from each technical replicate of each sample were calculated from each sample, and their means and standard deviation are plotted using a bar plot that was generated using Microsoft Excel (Version 16.16.27).

**Statistical analysis**

A dilution series spanning eight orders of magnitude of pPVYhvedi1 for standard curve analyses were performed in three technical replicates for each qPCR assay run, while eight technical replicates were performed for the non-template control (NTC). Regression analysis was conducted to calculate the average slope and y-intercept of each standard curve, which were then used to determine the gene copy number per milligram of sample as previously described. The qPCR efficiency was evaluated to determine the reliability and accuracy of the qPCR assay. Additionally, a single-factor analysis of variance (ANOVA) was performed to assess the consistency of standard curves generated across different qPCR assay runs.

## Reagents

**Table:** 

**Kit/Reagent**	**Manufacturer, Catalog No.**	**Usage**
RNA *later* ™ Stabilization Solution	Invitrogen, cat. no. AM7020	RNA Preservation
TRIzol™ Reagent	Invitrogen, cat. no. 15596018	RNA Extraction
Direct-zol™ RNA Microprep Kit	Zymo Research, cat. no. R2060	RNA extraction
SuperScript ^TM^ IV First-Strand Synthesis System	Invitrogen, cat. no. 18091050	cDNA synthesis
NEBNext ^®^ Q5 ^®^ Hot Start HiFi PCR Master Mix	New England Biolabs, cat. no. M0543S	Generation of viral DNA standard
pCR™Blunt II-TOPO™ plasmid	Invitrogen, cat. no. 450245	Generation of viral DNA standard
Zyppy ^TM^ Plasmid Miniprep Kit	Zymo Research, cat. no. D4036	Generation of viral DNA standard
Power SYBR™ Green PCR Master Mix	Applied Biosystems™, cat. no. 4368577	Quantification of PVY by qPCR
**Primer**	**Manufacturer**	**Usage**
893xF [5' TGAATGGGCTTATGGTTTGGTGC 3']	Integrated DNA Technologies	qPCR primer for absolute quantification of PVY
PVY-1 RP [5' ATATACGCTTCTGCAACATCTGAGA 3']	Integrated DNA Technologies	qPCR primer for absolute quantification of PVY
**Plasmid**	**Plasmid repository**	**Usage**
pPVYhvedi1	Addgene (plasmid #23464)	Cloned PVY plasmid for for qPCR
